# RuNi/TiZr-MMO Catalysts Derived from Zr-Modified NiTi-LDH for CO-Selective Methanation

**DOI:** 10.3390/molecules29143309

**Published:** 2024-07-13

**Authors:** Zhihui Li, Jiteng Ma, Xinfa Dong

**Affiliations:** Guangdong Provincial Key Laboratory of Green Chemical Product Technology, School of Chemistry and Chemical Engineering, South China University of Technology, Guangzhou 510640, China; 202211085598@mail.scut.edu.cn (Z.L.); 202020123659@mail.scut.edu.cn (J.M.)

**Keywords:** CO-selective methanation, H_2_-rich gas purification, Zr-promoted LDH, MMO, RuNi catalyst

## Abstract

CO-selective methanation (CO-SMET) is an efficient hydrogen-rich (H_2_-rich) gas purification technology for proton exchange membrane fuel cells. It is vital to develop suitable catalysts with good low-temperature activity for CO-SMET reactions. In this study, RuNi/TiZr_x_-mixed metal oxide (RuNi/TiZr_x_-MMO) catalysts with different molar ratios of Zr/Ti, derived from a Zr-promoted NiTi-layered double hydroxide (NiTi-LDH) precursor were successfully prepared using the co-precipitation and wet impregnation methods. The RuNi/TiZr_0.2_-MMO catalyst possesses higher catalytic performance in a lower temperature window of 180–280 °C, which can reduce the CO concentration to be below 10 ppm. The characterization results obtained from XRD, BET, SEM, TEM, XPS, TPR, and TPD suggest that the addition of ZrO_2_ increases the surface area of the catalyst, improves the dispersion of metallic nanoparticles, increases the reducibility of Ni species on the RuNi/TiZr_0.2_-MMO catalyst’s surface, and enhances the adsorption and activation ability of CO, resulting in remarkable catalytic performance at lower reaction temperatures. Moreover, the RuNi/TiZr_0.2_-MMO catalyst demonstrated long-term catalytic stability and carbon resistance.

## 1. Introduction

As society develops, the demand for energy is growing at an astonishing rate. The excessive use and development of traditional fossil fuels have caused irreversible damage to our environment. There is an urgent need to find environmentally friendly energy sources to replace traditional fossil fuels [1,2,3]. Proton exchange membrane fuel cells (PEMFCs), powered by hydrogen or hydrogen-rich fuels, have garnered significant attention due to their high energy yield, low operating temperature, and good stability performance, positioning them as promising and effective power sources [4,5,6]. In this category, a hydrogen-rich gas, which inevitably contains nearly 0.5–2% carbon monoxide (CO), is typically produced through steam reforming processes. Thus, it is necessary to thoroughly purify the hydrogen-rich gas to remove CO before feeding the fuel to the PEMFCs, as it may otherwise cause the severe deactivation of the anode catalyst due to CO poisoning [7]. CO-selective methanation (CO-SMET) and CO-preferential oxidation (PROX) are two feasible methods for deep CO removal. The PROX process relies on an external air or oxygen gas supply, whereas the CO-SMET process directly converts CO into CH_4_ without additional reactants [8,9]. The key part of CO-SMET is to develop the catalysts with high activity and selectivity at a lower reaction temperature.

Ni- and Ru-based catalysts have been widely used in CO-SMET [10,11,12,13]. Many support materials, such as metal oxides, carbon materials, metal organic frameworks (MOFs), and zeolite, have been investigated [14,15,16,17]. The mixed metal oxides (MMOs) derived from layered double hydroxides (LDHs) exhibit high metal dispersion, an enhanced specific surface area, and better thermal stability. MMOs have become a promising catalyst material and have attracted significant attention from researchers [18,19]. However, the agglomeration of metal particles often occurs in the process of deriving MMOs from LDHs, which may reduce the activity of the catalyst on the CO-SMET reaction [20,21]. Studies have shown that the addition of ZrO_2_ can effectively prevent the agglomeration of metal particles and significantly improve the activity of the catalyst at low temperatures [22,23]. Zhan et al. [24] found that Zr species play a crucial role in the formation of ZrO_2_-doped Ni/Al_2_O_3_ catalysts, which led to the formation of smaller metallic Ni particles. Wang et al. [25] reported that the utilization of ZrO_2_ as a promoter of the Ni/SiO_2_ catalyst notably enhances the catalytic activity, facilitating the complete transformation of CO into CH_4_ in a hydrogen-rich gas stream. Lu et al. [26] found that nickel catalysts that were supported on ZrO_2_-modified clay exhibited excellent catalytic performance for the methanation of CO due to the highly dispersed ZrO_2_ on the surface of the clay fragments, which helps prevent the sintering of Ni species, avoid the formation of spinel-phase NiAl_2_O_4_, and inhibit the occurrence of carbon deposition. In addition, ZrO_2_, as a promoter of a CO-SMET catalyst, can also enhance the adsorption and activation ability of CO, thereby increasing its CO-SMET activity [27].

In this study, a series of RuNi/TiZr_x_-MMO catalysts, derived from a NiTiZr_x_-LDH precursor with different molar ratios of Zr/Ti, were synthesized. The RuNi/TiZr_0.2_-MMO exhibited high performance of CO-SMET in a low reaction temperature range of 180–280 °C. The effect of the Zr on the LDH structure and the dispersion of Ni, as well as the characteristics of the catalyst, and so on, were investigated by means of XRD, BET, SEM, TEM, XPS, TPR, and TPD.

## 2. Results and Discussion

The XRD patterns of NiTi-LDH and NiTiZr_x_-LDH precursors with different molar ratios of Zr/Ti are presented in Figure S1. It can be seen that all the samples exhibit the characteristic peaks of hydrotalcite-like compounds at 11.3°, 22.85°, 34.37°, 38.72°, 60°, and 61.5° (PDF #15-0087), which are related to the reflection planes at (003), (006), (101), (105), (110), and (113), respectively [28,29]. Compared with NiTi-LDH, the peak intensity of the NiTiZr_x_-LDH (x = 0.1, 0.2, 0.3, 0.5) precursors decreased, indicating that the crystallinity of LDH can be decreased by the addition of ZrO_2_.

The nitrogen (N_2_) adsorption–desorption curves and pore size distributions of the NiTiZr_x_-LDH (x = 0.1, 0.2, 0.3, 0.5) precursors are shown in Figure S2. All the N_2_ adsorption–desorption isotherms of the NiTi-LDH and NiTiZr_x_-LDH precursors exhibited the typical IUPAC type IV curves and H3 hysteresis loops, indicating that they had a mesoporous and sheet-like structure [30,31]. The specific surface area and average pore size of all the precursors are summarized in Table S1. Obviously, with the increase in the molar ratio of Zr/Ti, the specific surface area of NiTiZr_x_-LDH increased. When the molar ratio of Zr/Ti is 0.2, the specific surface area of NiTiZr_x_-LDH reached the maximum value. However, when Zr/Ti was further increased, the specific surface area of NiTiZr_x_-LDH decreased. This suggests that the most suitable Zr/Ti ratio is 0.2. In this work, NiTiZr_0.2_-LDH was selected as the precursor of the catalyst support, and NiTi-LDH was used as the comparable sample.

**Figure 1:**
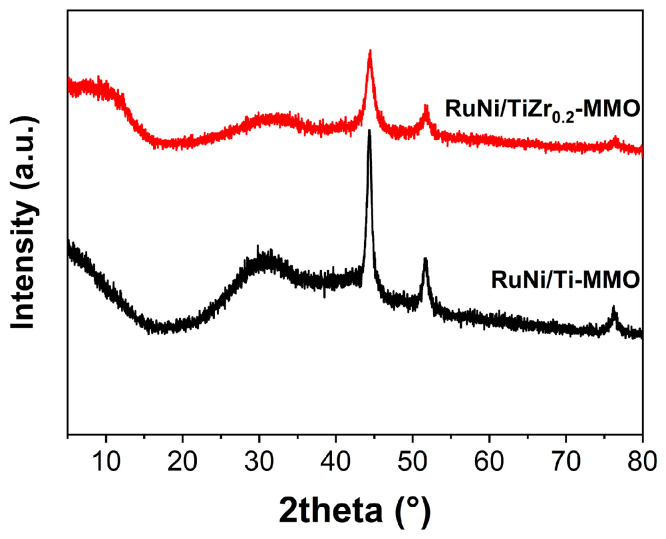
XRD patterns of the RuNi/Ti-MMO and RuNi/TiZr_0.2_-MMO catalysts.

The XRD patterns of the reduced samples of RuNi/Ti-MMO and RuNi/TiZr_0.2_-MMO are displayed in Figure 1. The characteristic peaks of metallic Ni were observed at 44.5°, 51.8°, and 76.4° (PDF #04-0850), which were related to the reflection planes at (111), (200), and (220), respectively [32]. There was no significant difference in the two-theta values in the diffraction peak of Ni between the two samples. However, compared with the RuNi/Ti-MMO, the intensity of the characteristic Ni peaks of the RuNi/TiZr_0.2_-MMO was significantly reduced. The size of the Ni for the NiTiZr_0.2_-MMO catalyst, which was estimated using the Scherrer equation [33] and is summarized in Table S2, was significantly smaller than that for the NiTi-MMO catalyst, indicating that the addition of a suitable amount of ZrO_2_ was beneficial for the dispersion of the Ni, possibly due to the structural effects of the ZrO_2_ promoter [27]. Thus, the RuNi/TiZr_0.2_-MMO catalyst achieves a better performance for the CO methanation reaction. In addition, no peaks of Ru were detected in the reduced samples, suggesting high dispersion and a small crystallite size [18]. Moreover, no peaks attributed to TiO_2_ and ZrO_2_ were observed in the XRD pattern of the samples, indicating that the catalyst support was composed of the amorphous structure of the oxides mixture (TiO_2_ and ZrO_2_) [23,34].

**Figure 2:**
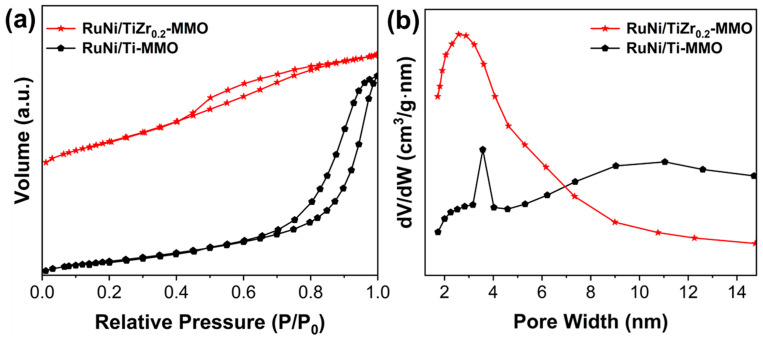
(**a**) N_2_ adsorption–desorption isotherms and (**b**) pore size distribution of the RuNi/Ti-MMO and RuNi/TiZr_0.2_-MMO catalysts.

The N_2_ adsorption–desorption isotherms of the RuNi/Ti-MMO and RuNi/TiZr_0.2_-MMO catalysts are shown in Figure 2a. The RuNi/Ti-MMO exhibited the typical IUPAC type IV curves and H3 hysteresis loop, indicating that the pore structure is very irregular; the RuNi/TiZr_0.2_-MMO exhibited the typical IUPAC type IV curves and H2 hysteresis loop, demonstrating that the pore size distribution is relatively uniform [31]. As shown in Figure 2b, RuNi/Ti-MMO exhibited a dual pore size distribution, with the main pore size distribution being around 3.5 nm, and an additional pore size distribution of 5–13 nm. After loading ZrO_2_, the pore sizes of RuNi/TiZr_0.2_-MMO are mainly distributed at around 3.0 nm. The Brunauer−Emmett−Teller (BET) surface area and average pore size of the RuNi/Ti-MMO and RuNi/TiZr_0.2_-MMO catalysts are summarized in Table 1. It is well known that a large specific surface area usually means more active sites in catalytic reactions [35]. Compared with RuNi/Ti-MMO (90 m^2^·g^−1^), the BET surface area of the RuNi/TiZr_0.2_-MMO catalyst (191 m^2^·g^−1^) increased significantly, which facilitates the dispersion of the active metal particles and thus improves the performance of the catalyst during CO methanation.

**Figure 3:**
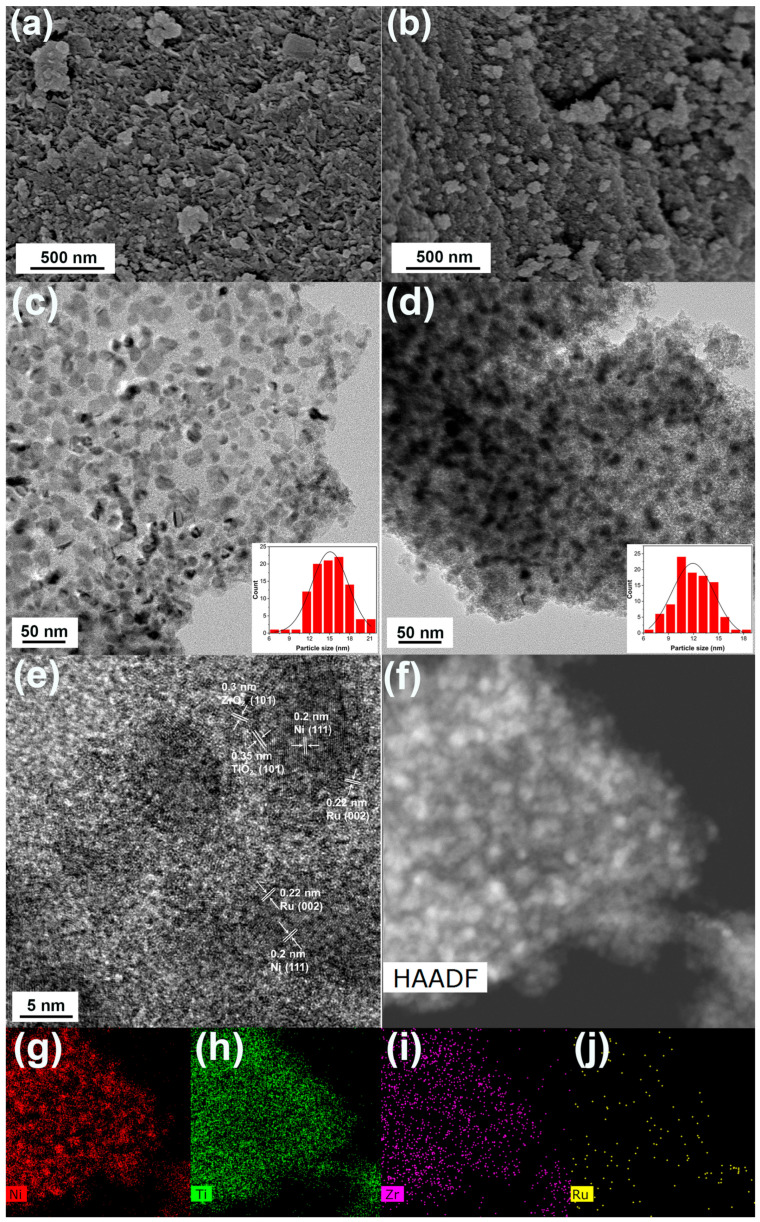
SEM images of (**a**) RuNi/Ti-MMO and (**b**) RuNi/TiZr_0.2_-MMO, (**c**) a TEM image of RuNi/Ti-MMO, (**d**) TEM, (**e**) HRTEM, and (**f**–**j**) HAADF-STEM and EDX elementary mapping images of RuNi/TiZr_0.2_-MMO.

The SEM and TEM images of the RuNi/Ti-MMO and RuNi/TiZr_0.2_-MMO catalysts are shown in Figure 3. As shown in Figure 3a, the RuNi/Ti-MMO exhibits a flake particles morphology. For the RuNi/TiZr_0.2_-MMO, the morphology consists of many aggregated nanoparticles, which can be seen in Figure 3b, indicating that the hydrotalcite-like structure of the LDH precursor was disrupted during the reduction process. As presented in Figure 3c,d, the Ni and Ru nanoparticles exhibited slight agglomeration on the Ti-MMO support, whereas on the TiZr_0.2_-MMO support, they were evenly distributed, without significant agglomeration. The distributions of the Ni and Ru nanoparticles on the RuNi/Ti-MMO and RuNi/TiZr_0.2_-MMO catalysts are presented as insets in Figure 3c,d. The average sizes of the Ni and Ru nanoparticles for the RuNi/Ti-MMO and RuNi/TiZr_0.2_-MMO catalysts are around 15 nm and 12 nm, respectively. In other words, the size of the Ni and Ru crystallites decreased with the addition of ZrO_2_, indicating that the introduction of ZrO_2_ can help reduce the size of the Ni and Ru nanoparticles, thereby significantly enhancing the catalytic activity of RuNi/TiZr_0.2_-MMO, which is in accordance with the XRD results. As shown in Figure 3e, the lattice fringes at 0.35 nm, 0.3 nm, 0.2 nm, and 0.22 nm are ascribed to the (101) plane of TiO_2_, the (101) plane of ZrO_2_, the (111) plane of Ni, and the (002) plane of Ru, respectively [23,36,37]. Figure 3f–j presents the high-angle annular dark-field scanning transmission electron microscopy (HAADF-STEM) and EDX elementary mapping images of RuNi/TiZr_0.2_-MMO. The results obtained by means of EDX mapping suggest that the Ni, Ti, Zr, and Ru elements were well distributed in the catalyst, which matches well with the TEM results.

**Figure 4:**
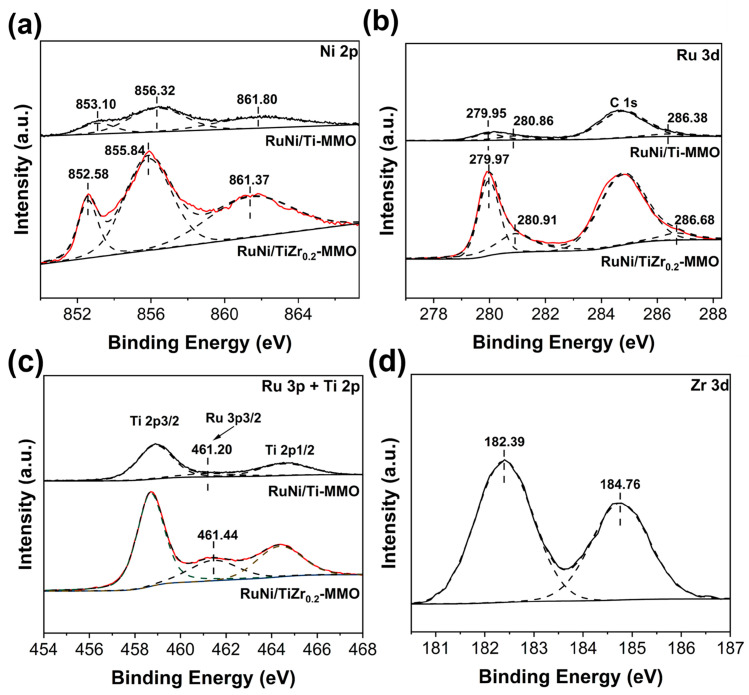
XPS spectra of (**a**) Ni 2p, (**b**) Ru 3d, and (**c**) Ru 3p and Ti 2p curves for the RuNi/Ti-MMO and RuNi/TiZr_0.2_-MMO catalysts. (**d**) XPS spectra of the Zr 3d curve of the RuNi/TiZr_0.2_-MMO catalyst.

The XPS spectroscopy was used to further characterize the surface chemical composition and oxidation states of RuNi/Ti-MMO and RuNi/TiZr_0.2_-MMO, as shown in Figure 4. For RuNi/Ti-MMO, two peaks were observed at about 853.10 eV and 856.32 eV in the Ni 2p XPS spectrum, which corresponds to the binding energies of Ni^0^ species and Ni^2+^ species, respectively, and the peak at 861.80 eV was assigned to the oscillating satellite peak [34]. The Ru 3d XPS spectrum is deconvoluted to three components occurring at 279.95 eV, 280.86 eV, and 286.38 eV, which are assigned to the metallic Ru^0^ 3d_5/2_, Ru^4+^ 3d_5/2_, and RuO_x_/Ru [38]. The Ru 3d_3/2_ peak is centered at approximately 284.80 eV, which completely overlaps with the C1s signal [39]. As shown in Figure 4c, the two main spectral signals with binding energies at 458. 71 eV and 464.37 eV belong to the Ti 2p_3/2_ and Ti 2p_1/2_ of Ti^4+^, respectively [38]. In addition, the signal located at 461.20 eV is attributed to Ru^0^ 3p_3/2_ in the Ru^0^ state, indicating the existence of metallic Ru in the RuNi/Ti-MMO samples [40]. Compared with RuNi/Ti-MMO, the characteristic peak of Ni in RuNi/TiZr_0.2_-MMO shifts to a lower binding energy, while the Ru peak shifts to a higher binding energy, indicating that more electrons are transferred from Ru to Ni, which can weaken the connection of the C-O bond of the CO that is adsorbed on the Ni surface due to the addition of the electron promoter ZrO_2_ species and thus promote CO dissociation in CO-SMET [41,42]. Figure 4d presents the Zr 3d XPS spectrum for the RuNi/TiZr_0.2_-MMO catalyst. The two major peaks at 182.39 eV and 184.76 eV belong to the Zr 3d_5/2_ and Zr 3d_3/2_ of Zr^4+^ species, respectively [27].

**Figure 5:**
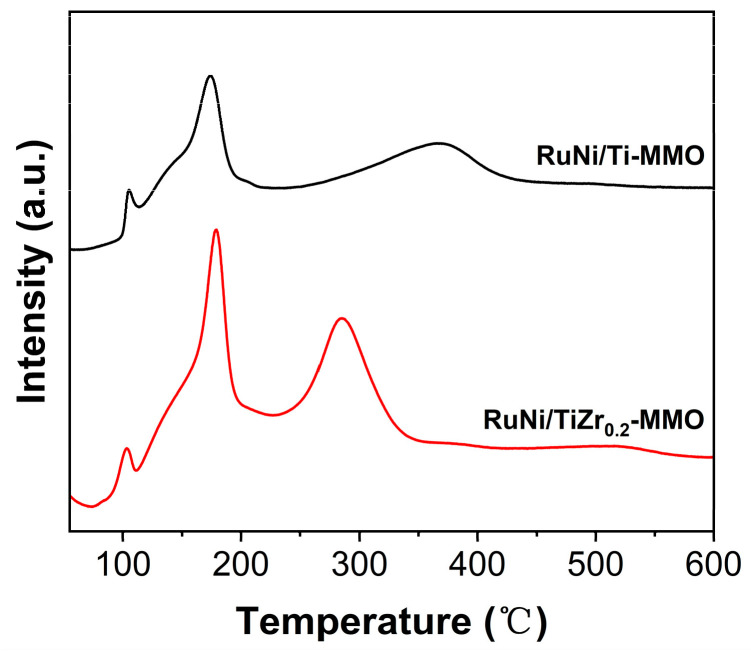
H_2_-TPR profiles of the RuNi/Ti-MMO and RuNi/TiZr_0.2_-MMO catalysts.

The H_2_-TPR profiles of the RuNi/Ti-MMO and RuNi/TiZr_0.2_-MMO catalysts are displayed in Figure 5. It can be seen that three reduction peaks exist in the range of 50–600 °C for the RuNi/Ti-MMO catalyst. The first peak, located at about 106 °C, was attributed to the reduction of Ru^3+^ to metallic Ru, and the second peak, located at about 174 °C, was assigned to the reduction of Ru^4+^ in ruthenium dioxides interacting with the MMO support [36]. The third peak, located at about 366 °C, was ascribed to the reduction of NiO [37]. It is well known that the reduction temperature mainly depends on the size and/or location of nanoparticles [43]. Compared with the RuNi/Ti-MMO catalyst, the third reduction peak for the RuNi/TiZr_0.2_-MMO catalyst shifted from 366 °C to 286 °C, suggesting that the addition of ZrO_2_ may be beneficial to the reduction of NiO, thereby contributing to the increase in the number of active Ni species sites and the decrease in the size of metallic nanoparticles. Moreover, in this study, the catalyst’s reduction temperature was 350℃, and NiO could be fully reduced in the RuNi/TiZr_0.2_-MMO catalyst, while NiO could only be partially reduced in the RuNi/Ti-MMO catalyst. Therefore, the RuNi/TiZr_0.2_-MMO catalyst showed better CO-SMET activity after reduction.

**Figure 6:**
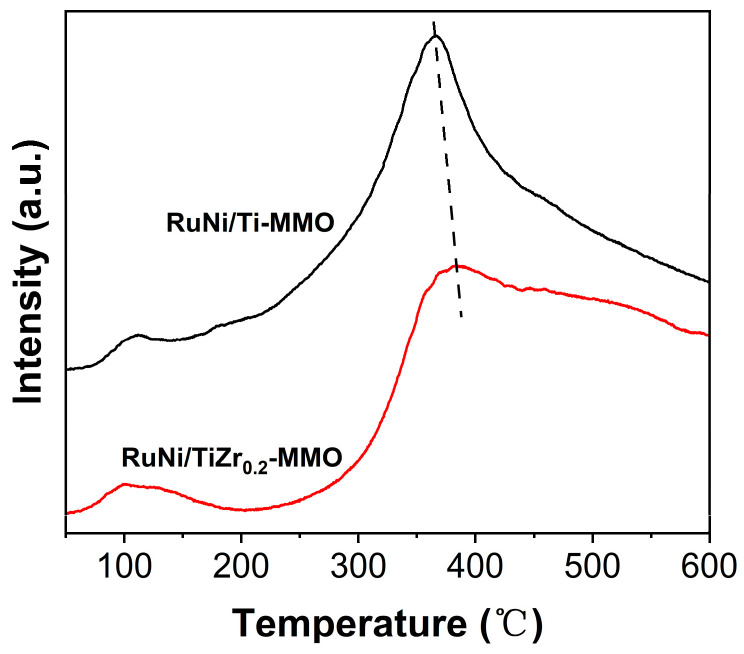
CO-TPD profiles of the RuNi/Ti-MMO and RuNi/TiZr_0.2_-MMO catalysts.

The CO-TPD profiles of the RuNi/Ti-MMO and RuNi/TiZr_0.2_-MMO catalysts are shown in Figure 6. Two peaks of CO desorption can be clearly observed. For the RuNi/Ti-MMO catalyst, the CO desorption peaks at temperatures of 109 °C and 366 °C were assigned to the desorption of single-site CO chemisorption and bridge CO chemisorption, respectively [44,45]. It is universally acknowledged that bridged chemisorption significantly contributes to the formation of CH_4_ compared with single-site chemisorption [46]. Compared with the RuNi/Ti-MMO catalyst, the desorption peaks of the bridge-adsorption CO for the RuNi/TiZr_0.2_-MMO catalyst shifted from 366 °C to 383 °C, suggesting that the addition of the ZrO_2_ enhanced the interaction between the CO and the active sites on the catalyst’s surface and, furthermore, guaranteed the activation of the CO, thereby promoting the CO-SMET reaction [47,48].

**Figure 7:**
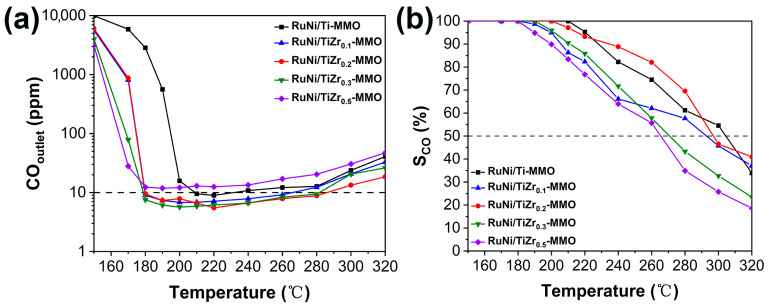
The outlet CO concentration (**a**) and the selectivity of CO methanation (**b**) over the RuNi/Ti-MMO and RuNi/TiZr_x_-MMO (x = 0.1, 0.2, 0.3, 0.5) catalysts.

Figure 7 shows the catalytic performance of the RuNi/Ti-MMO and RuNi/TiZr_x_-MMO catalysts towards CO-SMET. In this work, the suitable operating temperature window for CO-SMET was defined as the CO outlet concentration being below 10 ppm and the reaction selectivity being greater than 50% [23]. It was obvious that when the reaction temperature was at 180–200 °C, the CO-SMET reaction on all catalysts except RuNi/Ti-MMO and RuNi/TiZr_0.5_-MMO could reduce the CO outlet concentration to be below 10 ppm. Then, the CO outlet concentration gradually increased as the reaction temperature rose, due to the CO_2_-competitive reaction [16]. It is well known that low temperatures are favorable to CO methanation, while high temperatures are favorable to CO_2_-competitive methanation, so the selectivity of CO-SMET will be reduced if the reaction temperature is too high [49]. Combined with Figure 7a,b, the suitable operating temperature window of the RuNi/Ti-MMO catalyst was 210–230 °C. Meanwhile, the suitable operating temperature window of the RuNi/TiZr_0.2_-MMO catalyst was 180–280 °C, which is significantly better than that of the RuNi/Ti-MMO sample. The significant improvement in catalytic performance in the CO-SMET reaction can be ascribed to the increased surface area of the catalyst, the better dispersion of Ni and Ru particles, the promoted reducibility of Ni species, and the enhanced adsorption/activation of CO on the surface of the RuNi/TiZr_0.2_-MMO catalyst. For the RuNi/TiZr_0.5_-MMO catalysts, the CO outlet concentration only decreased to 15 ppm, suggesting that the excess addition of ZrO_2_ may have covered the active Ni sites, resulting in lower catalytic activity. In addition, the RuNi/TiZr_0.2_-MMO catalyst exhibited superior performance compared with the Ni- and Ru-based catalysts that were reported previously (Table S3).

For comparison, Ni-Ti or Ni-Ti-Zr mixed hydroxides were synthesized via a normal co-precipitation method using NaOH only as a pH-adjustor, and then calcined to produce Ni-Ti or Ni-Ti-Zr mixed oxides, which were used as support to prepare RuNi/Ti-MMO(nor) and RuNi/TiZr_0.2_-MMO(nor) catalysts using an impregnation method. As shown in Figure S3, although they can also eliminate CO from hydrogen-rich gases to be less than 10 ppm via CO-SMET, the suitable operating temperature window of the RuNi/Ti-MMO(nor) and RuNi/TiZr_0.2_-MMO(nor) catalysts were 191–245 °C and 183–238 °C, respectively, which is significantly smaller than that of the RuNi/TiZr_0.2_-MMO catalyst.

**Figure 8:**
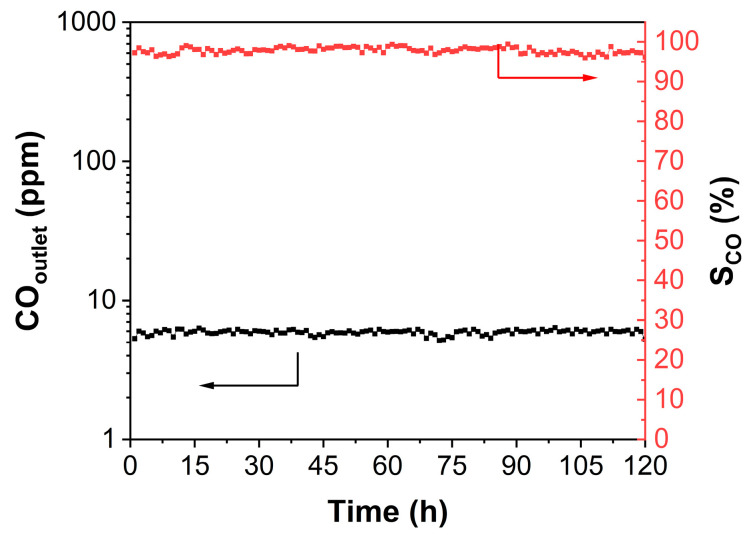
Long-term durability test of the RuNi/TiZr_0.2_-MMO catalyst at the temperature of 220 °C.

Figure 8 presents the long-term durability test at a reaction temperature of 220 °C, which is an important factor in determining whether the RuNi/TiZr_0.2_-MMO catalyst can be used as a CO-SMET catalyst. It can be seen that the outlet CO concentration of the RuNi/TiZr_0.2_-MMO catalyst is stable at about 6 ppm, and the reaction selectivity is always above 95% during the entire reaction time of 120 h.

**Figure 9:**
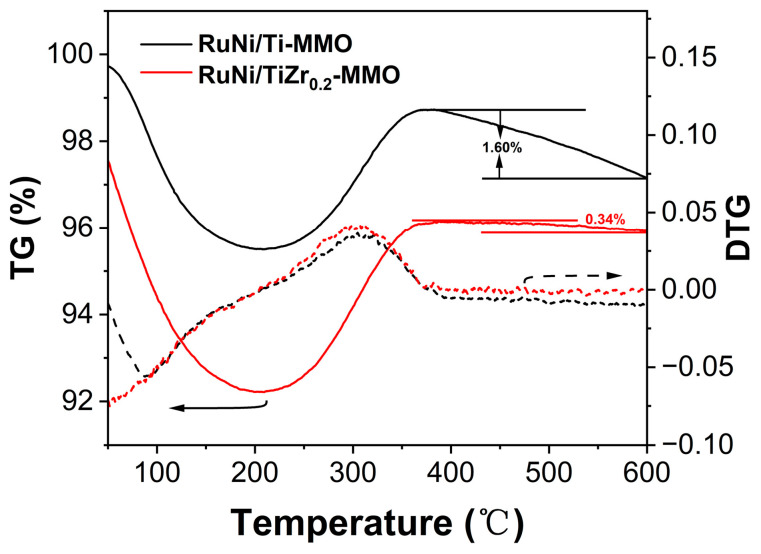
TG and DTG curves of the RuNi/Ti-MMO and RuNi/TiZr_0.2_-MMO catalysts.

Figure 9 illustrates the thermal decomposition of the spent catalysts of RuNi/Ti-MMO and RuNi/TiZr_0.2_-MMO after 10 h of CO-SMET reaction. As shown in Figure 9, the mass loss observed at temperatures below 200 °C can be ascribed to the removal of adsorbed water. The weight increase observed in the temperature window of 200 °C to 350 °C is attributed to the oxidation of metallic Ni [23]. The weight loss above 350 °C is due to the deposited carbon [50]. The weight loss rate of the spent RuNi/TiZr_0.2_-MMO (0.34%) was significantly lower than that of the spent RuNi/Ti-MMO (1.60%). The lower amount of deposited carbon of the spent RuNi/TiZr_0.2_-MMO indicated that the addition of a Zr promoter increased the catalyst’s resistance to the deposition of carbon; thus, the RuNi/TiZr_0.2_-MMO catalyst maintained good stability during the CO-SMET reaction.

## 3. Materials and Methods

### 3.1. Materials

All the chemicals and reagents, including RuCl_3_, Ni(NO_3_)_2_·6H_2_O, TiCl_4_, Zr(NO_3_)_4_∙5H_2_O, Na_2_CO_3_, NaOH, and H_2_O, are commercially available and were used as received.

### 3.2. Preparation of the Catalysts

**Figure 10:**
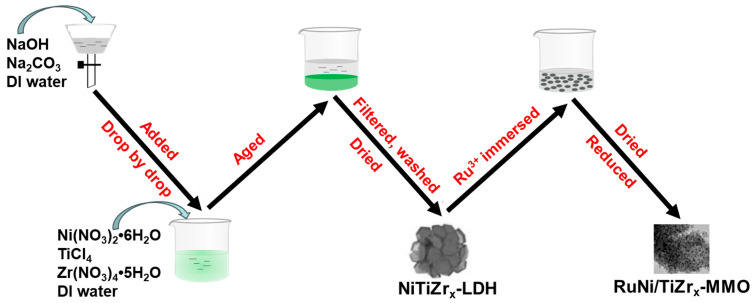
The schematic diagram showing the synthesis the RuNi/TiZr_x_-MMO (x = 0.1, 0.2, 0.3, 0.5) catalysts.

The schematic diagram showing the synthesis of the RuNi/TiZr_x_-MMO (x = 0.1, 0.2, 0.3, 0.5) catalysts is presented in Figure 10.

#### 3.2.1. Preparation of NiTi-LDH and NiTiZr_x_-LDH

The NiTiZr_x_-LDH precursors were synthesized by means of a co-precipitation method [51,52]. Firstly, appropriate amounts of Ni(NO_3_)_2_∙6H_2_O, TiCl_4_, and Zr(NO_3_)_4_∙5H_2_O were dissolved in 100 mL of deionized water and stirred vigorously for 0.5 h to form a 0.1 M solution A, in which the concentration of Ni^2+^ was 0.05 M and the concentrations of Ti^4+^ and Zr^4+^ were determined by the molar ratio of Zr/Ti. Appropriate amounts of Na_2_CO_3_ and NaOH were dissolved in 200 mL of deionized water to form a 2.5 M solution B. Subsequently, under stirring, solution B was added into solution A drop by drop until the pH value reached 9. Then, the obtained suspension was aged at 80 °C for 12 h. The sediment was filtered and washed with deionized water 3–5 times and dried at 60 °C to obtain the NiTiZr_x_-LDH precursors, where the x represented the Zr/Ti molar ratio (x = 0.1, 0.2, 0.3, 0.5). For comparison, pure NiTi-LDH was produced by means of the above procedure without the addition of Zr.

#### 3.2.2. Preparation of RuNi/Ti-MMO and RuNi/TiZr_x_-MMO

The Ru/NiTi-LDH or Ru/NiTiZr_x_-LDH samples were synthesized by means of the wet impregnation method. The Ru content of the prepared catalysts was maintained at 0.5 wt%. In a typical synthesis, 1 g of NiTi-LDH or NiTiZr_x_-LDH was immersed in 1.03 mL of 1 wt% RuCl_3_ solution for 24 h and dried at 60 °C overnight. Then, the Ru/NiTi-LDH or Ru/NiTiZr_x_-LDH samples were reduced at 350 °C under 50 vol.% H_2_/N_2_ flow of 60 mL·min^−1^ for 1.5 h and cooled down to room temperature under a N_2_ atmosphere to obtain the RuNi/Ti-MMO or RuNi/TiZr_x_-MMO catalysts (x = 0.1, 0.2, 0.3, 0.5).

### 3.3. Catalyst Characterization

X-ray diffraction (XRD) data were recorded using a Brucker D8 Advance diffractometer equipped with Cu Kα radiation (λ = 0.154 nm), operating at a voltage of 40 kV and a current of 40 mA to identify the structure of the crystal. The scanning range was set to between 5° and 80°. Prior to the test, 0.05 g of powdered sample was smeared uniformly onto a sample holder to ensure a flat upper surface. The Debye–Scherrer equation was used to calculate the Ni’s crystallite size.

The specific surface area and average pore size were confirmed using the Brunauer–Emmett–Teller (BET) method and the Barrer–Joyner–Halenda (BJH) equation on ASAP 2020. The nitrogen adsorption–desorption process was executed at −196 °C. Before the test, the sample catalyst was degassed in a vacuum at 80 °C for 12 h.

A German NETZSCH thermogravimetric analyzer (TG) was employed to study the thermal decomposition behaviors of the catalyst. Typically, 8 mg of the sample was weighed and placed in an Al_2_O_3_ ceramic crucible. Then, under an airflow of 20 mL·min^−1^, the temperature was incrementally increased from 50 °C to 600 °C at a heating rate of 10 °C·min^−1^, and the mass change curve of the sample was recorded.

Scanning electron microscopy (SEM) images were obtained using a Hitachi SU8220 scanning electron microscope at an accelerating voltage of 10 kV. Before the test, the sample was spread onto a conductive adhesive, and then, the conductive adhesive was stuck onto the sample table. The sample was subsequently sprayed with gold in the vacuum coating apparatus to enhance its conductivity.

Transmission electron microscopy (TEM) and high-resolution transmission electron microscopy (HRTEM) images were obtained using a JEOL JEM-2100F high-resolution transmission electron microscope at an accelerating voltage of 200 kV. Typically, the sample was uniformly dispersed in absolute ethanol for 10 min, and then, a small amount of suspension was applied to a copper grid coated with a carbon film. Subsequently, the sample was subjected to a drying treatment.

X-ray photoelectron spectra (XPS) were recorded using a Kratos Axis Ultra DLD Multifunctional photoelectron spectrometer equipped with a monochromatic Al Ka X-ray source (h*v* = 1486.6 eV). The binding energy of the carbon C 1s (284.8 eV) on the sample surface served as the internal standard. Before the test, 0.02 g powder samples were securely fixed on a sample stage using a conductive adhesive. The non-adhered powder was removed by gently blowing with an ear wash ball.

H_2_ temperature–programmed reduction (H_2_-TPR) was performed on a Micromeritics AutoChem II 2090 instrument equipped with a TCD detector. Prior to the test, 0.1 g of the sample was pretreated in a He flow at a heating ramp of 30 °C·min^−1^ at 300 °C for 0.5 h to purify its surface. After the sample was cooled down to 50 °C, the He flow was switched to a 10 vol% H_2_/Ar atmosphere at a flow rate of 30 mL·min^−1^, and the temperature was ramped from 50 °C to 600 °C at a heating rate of 10 °C·min^−1^. The signal of the H_2_ that was used for the catalyst reduction was detected.

CO temperature–programmed desorption (CO-TPD) was performed on a Microtrac BELCAT-A instrument equipped with a TCD detector. Firstly, 0.1 g of the catalyst was pre-reduced in a 50 vol% H_2_/N_2_ atmosphere with a flow rate of 60 mL·min^−1^ at 350 °C for 1.5 h. After cooling to room temperature with a N_2_ flow, a CO atmosphere with a flow rate of 30 mL·min^−1^ was introduced for 1 h until saturation. Then, the CO was switched to a He atmosphere at a flow rate of 30 mL·min^−1^ for 1 h to remove the weakly adsorbed CO on the surface of the catalysts. Lastly, the temperature was ramped from room temperature to 600 °C at a heating rate of 10 °C·min^−1^. The signal of the CO that was desorbed on the catalyst during this process was detected.

### 3.4. Catalyst Evaluation

The CO-selective methanation performance evaluation was performed with 0.2 g of catalysts (40–60 mesh) in a 6 mm diameter fixed-bed quartz tubular reactor at atmospheric pressure in the temperature range of 150–320 °C. The reactants (1 vol% CO, 20 vol% CO_2_, and 79 vol% H_2_) were co-fed into the reactor at a flow rate of 20 mL·min^−1^. Prior to the reaction, the catalysts were pretreated with 50 vol% H_2_/N_2_ at 350 °C for 1.5 h at a flow rate of 60 mL·min^−1^. The reaction temperature was measured and controlled using a thermocouple. The compositions of the feedstock and effluent gases were analyzed using an on-line Agilent 7820A gas chromatograph equipped with a flame ionization detector (FID) and a thermal conductivity detector (TCD). The catalytic activity was evaluated based on the CO concentration in the effluent [16]. The CO selectivity (S_CO_) of the catalyst was calculated by the following formula:

(1) $$S_{CO}=\frac{F_{CO}^{in}-F_{CO}^{out}}{F_{CH4}^{out}}\times 100\%$$

Here, $F_{CO}^{in}$ is the feedstock flow rate of CO, mmol·min^−1^; $F_{CO}^{out}$ is the effluent flow rates of CO, mmol·min^−1^. $F_{CH4}^{out}$ is the effluent flow rate of CH_4_, mmol·min^−1^.

## 4. Conclusions

In conclusion, a series of RuNi/TiZr_x_-MMO catalysts with different molar ratios of Zr/Ti, derived from a Zr-promoted NiTi-LDH precursor, were successfully prepared using the co-precipitation and wet impregnation methods. The CO in a hydrogen-rich gas can be removed to a level of less than 10 ppm using a RuNi/TiZr_0.2_-MMO catalyst under the suitable operating temperature window of 180–280 °C and with a selectivity of more than 50%. The addition of ZrO_2_ can increase the surface area of the catalyst, improve the dispersion of Ni and Ru nanoparticles on the catalyst, and greatly enhance the activity of the catalyst. Moreover, the addition of ZrO_2_ can also promote the reducibility of Ni species on the RuNi/TiZr_0.2_-MMO catalyst’s surface, enhance the adsorption capacity of the CO, promote the dissociation of the CO, and increase the catalyst’s resistance to the deposition of carbon, which can promote the CO-SMET reaction.

## Figures and Tables

**Table 1:** Specific surface area and average pore size of the RuNi/Ti-MMO and RuNi/TiZr_0.2_-MMO catalysts.

Catalyst	Surface Area (m^2^·g^−1^)	Average Pore Size (nm)
RuNi/Ti-MMO	90	7.4
RuNi/TiZr_0.2_-MMO	191	3.6

## Data Availability

The datasets generated and/or analyzed during the current study are available from the corresponding author upon reasonable request.

## Supplementary Materials

The following supporting information can be downloaded at: https://www.mdpi.com/article/10.3390/molecules29143309/s1, Figure S1: XRD patterns of NiTi-LDH and NiTiZr_x_-LDH (x = 0.1, 0.2, 0.3, 0.5) precursors; Figure S2: (**a**) N_2_ adsorption–desorption curves and (**b**) pore size distribution of NiTi-LDH and NiTiZr_x_-LDH (x = 0.1, 0.2, 0.3, 0.5) precursors; Figure S3: The outlet CO concentration (**a**) and the selectivity of CO methanation (**b**) over the RuNi/Ti-MMO(nor) and RuNi/TiZr_0.2_-MMO(nor) catalysts; Table S1: The specific surface area and average pore size of NiTi-LDH and NiTiZr_x_-LDH (x = 0.1, 0.2, 0.3, 0.5) precursors; Table S2: The size of the Ni estimated by the Scherrer equation of RuNi/Ti-MMO and RuNi/TiZr_0.2_-MMO; Table S3: Summary of the CO-SMET performances of the Ru- and Ni-based catalysts reported previously. References [18,34,37,53,54,55,56,57,58,59] are cited in the Supplementary Materials.
