# Supplementary material for: RuNi/TiZr-MMO Catalysts Derived from Zr-Modified NiTi-LDH for CO-Selective Methanation
Source: Molecules. 2024 Jul 13;29(14):3309. doi: 10.3390/molecules29143309 (PMC11279186; doi:10.3390/molecules29143309)
Supplement: Supplementary file 1 [file molecules-29-03309-s001.zip › molecules-3053811-Supplementary Materials.pdf]

Supplementary Materials

RuNi/TiZr-MMO Catalysts Derived from Zr-Modified NiTi-LDH for CO-Selective Methanation

Zhihui Li †, Jiteng Ma † and Xinfa Dong *

Guangdong Provincial Key Laboratory of Green Chemical Product Technology, School of Chemistry and Chemical Engineering, South China University of Technology, Guangzhou 510640, China; 202211085598@mail.scut.edu.cn (Z.L.); 202020123659@mail.scut.edu.cn (J.M.)

* Correspondence: cexfdong@scut.edu.cn

† These authors contributed equally to this work.

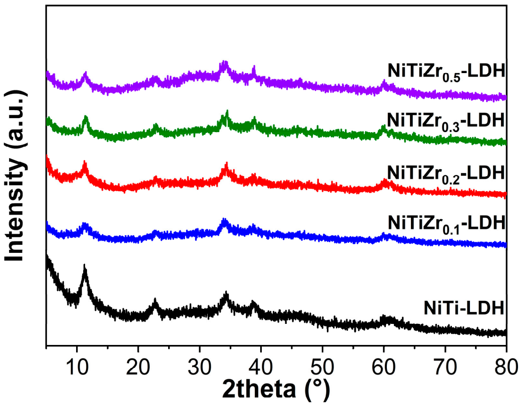

Figure S1. XRD patterns of NiTi-LDH and NiTiZrx-LDH (x = 0.1, 0.2, 0.3, 0.5) precursors.

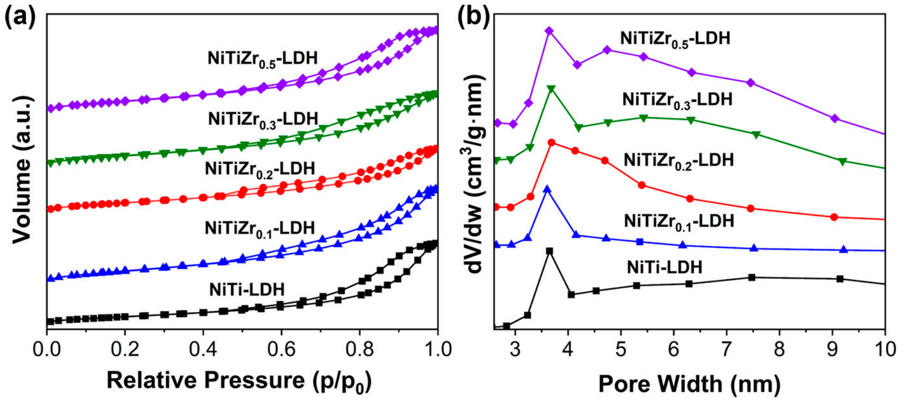

Figure S2. (a) N_2 adsorption–desorption curves and (b) pore size distribution of NiTi-LDH and NiTiZrx-LDH (x = 0.1, 0.2, 0.3, 0.5) precursors.

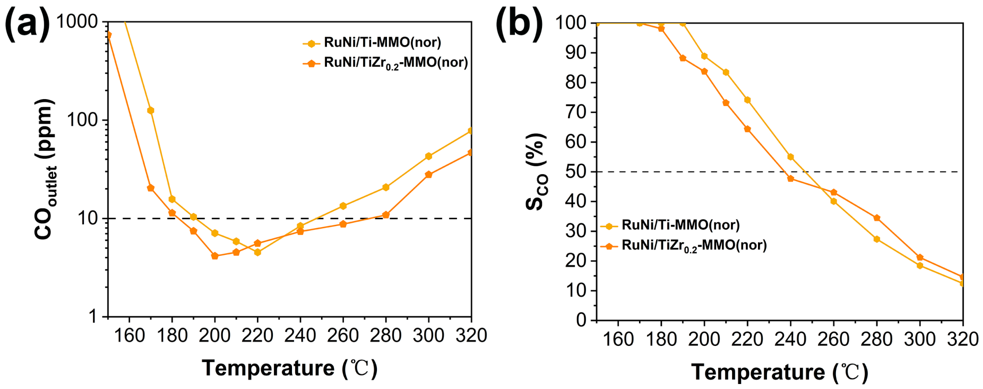

Figure S3. The outlet CO concentration (a) and the selectivity of CO methanation (b) over the RuNi/Ti-MMO(nor) and RuNi/$TiZr_{0.2}$-MMO(nor) catalysts.

Table S1. The specific surface area and average pore size of NiTi-LDH and $NiTiZr_x$-LDH (x = 0.1, 0.2, 0.3, 0.5) precursors.

Catalyst	Surface area ($m^2 \cdot g^{-1}$)	Average pore size (nm)
NiTi-LDH	288	11.6
$NiTiZr_{0.1}$-LDH	342	7.8
$NiTiZr_{0.2}$-LDH	441	8.9
$NiTiZr_{0.3}$-LDH	389	7.9
$NiTiZr_{0.5}$-LDH	411	8.5

Table S2. The size of the Ni estimated by Scherrer equation of RuNi/Ti-MMO and RuNi/$TiZr_{0.2}$-MMO.

Catalyst	D_{Ni} (nm)
RuNi/Ti-MMO	11.2
RuNi/$TiZr_{0.2}$-MMO	6.5

Table S3. Summary of the CO-SMET performances of the Ru- and Ni-based catalysts reported previously.

Catalyst	$[CO]_{min}$ (ppm)	SWTW [1]	Stability (h)	Reference
RuNi/$TiZr_{0.2}$-MMO	<10	180~280	120	In this work
Ru-Ni/Al_2O_3	<100	*	-	[18]
RuNi/MMO	<10	180~260	120	[34]
RuNi/GA-MMO	<10	220~300	120	[37]
NiAl-Na	-	240~320	100	[53]
Ru-Ni/Al_2O_3	11	*	80	[54]
Ni/TiO_2	1000	*	-	[55]
Ru-Ni/TiO_2	11	*	-	[56]
Ru-Ni/TiO_2-Al_2O_3	<10	210~220	200	[57]
Ru/Ni-TNTs	<10	210~285	-	[58]
Ru/Ni-Al-oxide/NF	<10	200~260	120	[59]

[1] A suitable working temperature window, in which the CO concentration in the effluent less than 10 ppm and CO selectivity greater than 50%.
* The catalyst did not meet the requirements.
